# Supplementary material for: Prevalence and risk factors of hypertension among civil servants in Sidama Zone, south Ethiopia
Source: PLoS One. 2020 Jun 11;15(6):e0234485. doi: 10.1371/journal.pone.0234485 (PMC7289366; doi:10.1371/journal.pone.0234485)
Supplement: S1 File — (PDF) [file pone.0234485.s001.pdf]

English version questionnaire

Hawassa University

College of Medicine and Health Sciences

School of Public Health

Questionnaire designed to study the prevalence and risk factors of hypertension among civil servants of Sidama Zone Administration

Respondent's code : ____________________

3. Interview questions

Section I: Socio-demographic characteristics

No	Questions	Response/Alternative Choices	Remark
101	What is the sex of the respondent?	1. Male<br>0. Female	
102	What is your age (in completed years)?	__________ years	
103	What is your current marital status? (encircle the response)	1. Single<br>2. Married<br>3. Divorced<br>4. Widowed	
104	What is your Religious affiliation? (encircle the response)	1. Protestant Christian<br>2. Orthodox Christian<br>3. Catholic<br>4. Muslim<br>5.Other (specify) __________	
105	To which ethnic group do you belong?	1. Sidama<br>2. Amhara<br>3. Oromo<br>4. Gurage<br>5. Wolayita<br>88.Other,specify_____	
106	What is your highest educational level you have completed?	1. Cannot read and write<br>2. Read and write only<br>3. Primary education(1-8)<br>4. Secondary education(9-12)<br>5. Diploma and above	
107	What is your current work position in this depatment/ office?	1.High level managerial work<br>2. Expert (professional)<br>3.Supportive staff (secretary/ janitor/guard/driver, etc	
108	What is your family size? (In number)	________	
109	What is your family's average monthly income?	Approximate_____________ ETB<br>99. Don't know	

Section II. Knowledge of respondents regarding hypertension prevention

No	Questions	Response / Alternative Choices	Code
201	Have you ever heard about hypertension? (If no skip to question no. 301)	1. yes<br>0. no	
202	Is hypertension a transmissible disease?	1. yes<br>0. no	
203	Can hypertension be prevented?	1 yes<br>0. no	
204	If yes to question # 203, what methods do you know to prevent hypertension? (more than one answer is possible)	1. Avoid smoking<br>2. Avoid drinking excessive amount of alcohol<br>3.Doing regular physical exercise<br>4.Consuming fruits and vegetable and reducing consumption of excess fat<br>5. Restricting sodium consumption<br>6.Other, specify________________	
205	Have you ever received any information on Hypertension from health workers?	1. yes<br>2. no<br>3. I do not remember	
206	Do you know where to get measured for blood pressure?	1 yes<br>2. no	
207	Do you know the normal blood pressure value?	1 Yes<br>2. No	
208	If "yes" to Q#207, Ask the respondent for the normal value (check if he/she say 120/80)	_______________	
209	Do you know the risk behavior for contracting hypertension	1. yes<br>2. no	
210	If yes to question # 209, what are the risk behaviors for contracting hypertension?<br>(more than one answer is possible)	1. Cigarette smoking<br>2. Physical inactivity<br>3. Excessive alcohol consumption<br>4. Eating animal fat frequently<br>5 excess salt Consumption<br>6 Other, specify ____________	

Section III. Behavioral Measurements

**Cigarette use**			
No.	Question	Response / Alternative choices	Remark
301	Have you ever smoked any cigarette products like cigars or pipes?<br>If the answer is “no” , skip to Q # 306	1. yes<br>0. no	
302	Did you smoke in the past 30 days prior to this survey?	1. yes<br>0. no	
303	If “yes” for Q 302 how frequent do you smoke in a week?	1. daily<br>2. 5-6 days<br>3. 3-4 days<br>4. 1-2 days	
304	For how long have you smoked cigarette?	______ years	
305	On average, how many cigarettes do you smoke each day?	____________ (in number)	
**Alcohol Consumption**			
306	Have you ever consumed alcoholic drink? (“tella”,” tej”, “Araki”, beer, wine, spirit)<br><br>If the answer is “no” , skip to Q #311	1.yes<br>0. no	
307	Did you drink alcohol in the past 30 days prior to this survey?	1. Yes<br>2. no	
308	If yes for Q307 how frequent do you drink alcohol in a week?	1. daily<br>2. 5-6 days per week<br>3. 3-4 days per week<br>4. 1-2 days per week	
309	For how long have you been drinking?	___________(in year)	
310	How many glass/ bottle do you drink at a time? (specify the type of drink)	___________	
**Khat chewing**			
311	Have you ever chewed khat?<br>If the answer is “no” , skip to Q #411	1.yes<br>0.no	
312	Did you chew khat in the past 30 days prior to this survey?	1.yes<br>0. no	
313	If yes for Q 312 how frequent do you chew?	1. daily	

		2. 5-6 days per week<br>3. 3-4 days per week<br>4. 1-2 days per week	
314	For how long have you been chewing chat?	__________(in years)	
**Caffeine Consumption**			
315	Do you drink coffee?<br>(if "no" skip to Q number 318)	1. yes<br>0. no	
316	How many days in a week do you drink coffee?	1. daily<br>2. 5-6 days in a week<br>3. 3-4 days in a week<br>4. 1-2 days in a week	
317	How many cup of coffee do you drink in these days?	1. one cup a day<br>2. two cups a day<br>3. three and more cups a day	
**Diet**			
318	How many days do you eat fruits in a week?	______Number of days	
319	How many servings of fruits do you eat in these days?	______Number of servings	
320	How many days do you eat vegetables in a week?	______Number of days	
321	How many servings of vegetables do you eat in these days?	______Number of servings	
322	Do you eat animal fat (butter, fatty meat)?	1. yes<br>0. no	
323	How many days do you eat animal fat in a week?	______Number of days	
324	How many servings of animal fat do you eat in these days?	______Number of servings	
**Salt consumption**			
325	Do you use salt in your food?	1. yes<br>0. no	
326	Do you use additional top added salt on plate after food is prepared with sufficient amount of salt?	1. yes<br>0. no	
**Physical activity**			
327	Does your work involve vigorous-intensity activity that causes large increases in breathing or heart rate like (carrying or lifting heavy loads, digging or construction work) for at least 10 minutes continuously?	1. yes<br>0. no	
328	In a typical week, on how many days do you do vigorous-intensity activities as part of your work?	_____ days	
329	How much time do you spend doing	______ hours	

	vigorous-intensity activities at work on a typical day?		
**Travel to and from places**			
330	Do you walk for at least 10 minutes a day continuously to get to and from places?	1. yes<br>0. no	
331	In a typical week, for how many days do you walk for at least 10 minutes continuously to get to and from places?	______ days	
332	How much time do you spend walking for travel on a typical day?	______ hours<br>______ minutes	
333	What do you use to go and come from place to place?	1.on foot<br>2. bicycle<br>3. engine using vehicle	
**Recreational activities**			
334	Do you do any vigorous-intensity sports, fitness or recreational (leisure) activities that cause large increases in breathing or heart rate like [running or football] for at least 10 minutes continuously?	1. yes<br>0. no	
335	In a typical week, for how many days do you do vigorous-intensity sports, fitness or recreational (leisure) activities?	______ days	
336	How much time do you spend doing vigorous-intensity sports, fitness or recreational activities on a typical day?	______ minutes	

Section IV. History of Raised Blood Pressure and co-morbidity

401	Have you ever had your blood pressure measured by a doctor or other health worker?	1. yes<br>0. no	
402	Have you ever been told by a doctor or other health worker that you have hypertension?	1. yes<br>0. no	
403	Are you currently receiving any medication, treatments/advice for hypertension prescribed by a doctor or other health worker?	1. yes<br>0. no	
404	Is there anyone from your family (father, mother or siblings) who have history of hypertension	1. yes<br>0. no	
405	Have you ever been told by a doctor or health worker that you have diabetes?	1. yes<br>0. no	

Section V. Physical Measurements

**Height and Weight**

No.	Question	Measurements	Remark
501	Height in cm	________cm	
502	Weight in kg	_______kg	
503	Hip circumference	________cm	
504	Waist circumference	________cm	
**Blood Pressure**			
505	Reading 1	_____Systolic (mmHg)	
		_____Diastolic (mmHg)	
506	Reading 2	_____Systolic (mmHg)	
		_____Diastolic (mmHg)	
507	Reading 3	_____Systolic (mmHg)	
		_____Diastolic (mmHg)	
