# Supplementary material for: Prevalence and risk factors of hypertension among civil servants in Sidama Zone, south Ethiopia
Source: PLoS One. 2020 Jun 11;15(6):e0234485. doi: 10.1371/journal.pone.0234485 (PMC7289366; doi:10.1371/journal.pone.0234485)
Supplement: S2 File — (PDF) [file pone.0234485.s002.pdf]

Amharic-version questionnaire

የስምምነት ቅጽ

ሀዋሳ ዩኒቨርሲቲ

የሕክምናና ጤና ሳይንስ ኮሌጅ

የኅብረተሰብ ጤና ት/ቤት

ይህ ቃለ መጠይቅ በሲዳማ ዞን አስተዳደር የመንግስት ሰራተኞች የደም ግፊት በሽታ ስርጭትና አጋላጭ መንስኤ ሊሆኑ የሚችሉ ምክንያቶችን ለማጥናት የተዘጋጀ ነዉ፡፡

የጥናቱ ተሳታፊ መለያ ቁጥር / ኮድ ---------------------

ክፍል አንድ፡ ሶሽዮ-ዴሞግራፊክ ሁኔታ			
ቁጥር	ጥያቄዎች	ምላሽ	ምርመራ
101	የመላሹ ጾታ ምንድን ነዉ?	1. ወንድ<br>0. ሴት	
102	እድሜዎ ስንት ነዉ? (በዓመት)	________ ዓመት	
103	የጋብቻ ሁኔታ? (ምላሹን ያክብቡ)	1. ያላገባ/ች<br>2. ያገባ/ች<br>3. ፍቺ የፈጸመ/ች<br>4. ባል/ሚስት በህይወት የሌለ	
104	ሃይማኖትዎ ምንድን ነዉ?	1. ፕሮቴስታንት<br>2. ኦርቶዶክስ<br>3. ካቶሊክ<br>4. ሙስሊም<br>5. ሌላ (ይግለጹ)......................	
105	የየትኛዉ ብሔር ተወላጅ ነዎት?	1. ሲዳማ<br>2. አማራ<br>3. ኦሮሞ<br>4. ጉራጌ<br>5. ወላይታ<br>6. ትግሬ<br>88. ሌላ (ይግለጹ)-----------------------	
106	ያጠናቀቁት ከፍተኛ የትምህርት ደረጃ የትኛዉ ነዉ?	1. መጻፍና ማንበብ የማይችል /የማትችል<br>2. መጻፍና ማንበብ ብቻ የሚችል /የሚትችል<br>3. አንደኛ ደረጃ (1-8)<br>4. ሁለተኛ ደረጃ (9-12)<br>5. ዲፕሎማ እና ከዚያ በላይ	
107	በዚህ መምሪያ/ ጽ/ቤት አሁን እየሠሩ ያሉበት የስራ መደብ የትኛዉ ነዉ?	1. ኃላፊ ወይም የሥራ ሂደት አስተባባሪ<br>2. ኤክስፐርት ወይም ባለሙያ<br>3. አስተዳደር ሠራተኛ (ጸሐፊ/ጽዳት/ጥበቃ/ ሾፌር …ወዘተ)	
108	የቤተሰብዎ ብዛት ስንት ነዉ? (በቁጥር)	_________	

109	ወርሃዊ የቤተሰብ አማካይ ገቢ ስንት ብር ነዉ ?	__________ብር<br>99. አላውቅም	

ክፍል ሁለት፡ የደም ግፊት በሽታን በመከላከል ላይ ተሳታፊዎች ያላቸዉ እዉቀት			
ቁጥር	ጥያቄ	ምላሽ	ምርመራ
201	ስለ የደም ግፊት በሽታ ሰምተው ያውቃሉ?<br><br>መልሱ "አዎ" ከሆነ ጥያቄ ቁ.202 ይጠይቁ ፤መልሱ "አላውቅም" ከሆነ ወደጥያቄ ቁ. 301 ይሂዱ	1. አዎ<br>0. አላውቅም	
202	የደም ግፊት በሽታ ተላላፊ በሽታ ነው ?	1. አዎ<br>0. አይደለም	
203	የደም ግፊት በሽታን መከላከል ይቻላል?	1. አዎ<br>0. አይደለም	
204	ለተራ ቁጥር 203 ምላሽዎ "አዎ" ከሆነ ምን አይነት መከላከያ ዘዴዎችን ያዉቃሉ(ከአንድ በላይ ምላሽ መመለስ ይቻላል)	1. ሲጋራ ማጨስ ማቆም<br>2.አልኮል ከመጠን በላይ መጠጣትን ማቆም<br>3. ተከታታይ የአካል ብቃት እንቅስቃሴ ማድረግ<br>4. አትክልትና ፍራፍሬ መመገብና ቅባት/ጮማ የበዛበትን ምግብ መመገብ መቀነስ<br>5. ጨዉ(ሶዲዬም) የበዛበትን ምግብ መመገብ መቀነስ<br>6.ሌላ ካለ ይጥቀሱ__________	
205	ስለ ደም ግፊት በሽታ ከጤና በለሙያዎች መረጃ ወይም ትምህርት አግኝተው ያውቃሉ?	1. አዎ<br>2.አልሰማሁም/አልተማርኩት<br>3. አላስታውስም	
206	የደም ግፊት መጠን ልኬት አገልግሎት የት እንደሚሰጥ ያውቃሉ?	1. አዎ<br>0. አላውቅም	
207	የተስተካከለ የደም ግፊት መጠን ስንት እንደሆነ ያውቃሉ?	1. አዎ<br>0. አላውቅም	
208	የጥያቄ ቁጥር 207 መልስ "አዎ" ከሆነ መጠኑ ስንት ነው?<br>(መላሹ 120/80 ማለታቸዉን ያረጋግጡ)	----------------------	

209	ለደም ግፊት በሽታ የሚያጋልጡ ባህሪያትን ያውቃሉ?	1. አዎ<br>0. አላውቅም	
210	የጥያቄ ቁጥር 209 መልስ "አዎ" ከሆነ ምን ምን ባህሪያት ለደም ግፊት በሽታ ያጋልጣሉ?<br><br>(ከአንድ በላይ መልስ መመለስ ይቻላል)	1. ሲጋራ ማጨስ<br>2. የአካል ብቃት እንቅስቃሴ ጉድለት<br>3. ከመጠን ያለፈ አልኮል መጠጣት<br>4. ጮማ/ ቅባት የበዛበትን ምግብ አዘወትሮ መመገብ<br>5. ብዙ ጨው መመገብ<br>6. ሌላ ካለ ይግለጹ __________	

**ክፍል ሶስት፡ የባህርይ መመዘኛዎች**			
**ሲጋራ አጠቃቀም**			
ቁጥር	ጥያቄ	ምላሽ	ምርመራ
301	ማንኛዉንም አይነት የሲጋራ ዉጤት (ፒፓ፣ሲጋራ ወዘተ) አጭሰው ያውቃሉ? መልሱ "አላውቅም" ከሆነ ወደ ጥያቄ ቁጥር 306 ይሂዱ	1. አዎ<br>0. አላውቅም	
302	ከዚህ ጥናት በፊት ባለፉት 30 ቀናት ዉስጥ ሲጋራ አጭሰዉ ያዉቃሉ?	1. አዎ<br>0. አላጨስኩም	
303	ለጥያቄ 302 ምላሽዎ "አዎ" ከሆነ በምን ያህል ጊዜ ውስጥ ያጨሳሉ?	1. በየቀኑ<br>2. በሳምንት ከ5-6 ቀናት<br>3. በሳምንት ከ3-4 ቀናት<br>4. በሳምንት ከ1-2 ቀናት	
304	ለምን ያህል ጊዜ አጭሰዋል?	______ አመት	
305	በአማካይ በቀን ምን ያህል ሲጋራ ያጨሳሉ?	______ በቁጥር	
**አልኮል አጠቃቀም**			
306	አልኮል ነክ መጠጦችን ጠጥተው ያውቃሉ? (ጠላ፣ጠጅ፣አረቂ፣ወይን፣ቢራ… ወዘተ) (መልሱ "አላውቅም" ከሆነ ወደ ጥያቄ ቁጥር 311 ይሂዱ)	1. አዎ<br>0. አላውቅም	
307	ከዚህ ጥናት በፊት ባለፉት 30 ቀናት ዉስጥ አልኮል ጠጥተዉ ያዉቃሉ?	1. አዎ<br>0. አልጠጣሁም	
308	ለጥያቄ 307 ምላሽዎ "አዎ" ከሆነ በሳምንት ስንት ቀን ነዉ አልኮል መጠጥ የሚጠጡት?	1. በየቀኑ<br>2. በሳምንት ከ5-6 ቀናት<br>3. በሳምንት ከ3-4 ቀናት	

		4. በሳምንት ከ1-2ቀናት	
309	ለምን ያህል ጊዜ ነዉ መጠጥ የጠጡት? (በአመት ይግለጹ)	______አመት	
310	በአንድ ጊዜ ምን ያህል ብርጭቆ/ጠርሙስ ይጠጣሉ? (በመጠጡ አይነት ይግለጹ)	______ በቁጥር	
**ጫት መቃም**			
311	ጫት ቅመው ያውቃሉ?<br><br>(መልሱ "አላውቅም" ከሆነ ወደ ጥያቄ ቁጥር 315 ይሂዱ)	1. አዎ<br>0. አላውቅም	
312	ከዚህ ጥናት በፊት ባለፉት 30 ቀናት ዉስጥ ጫት ቅመዉ ያዉቃሉ?	1. አዎ<br>0. አልቃምኩም	
313	ለጥያቄ ቁጥር 312 ምላሽዎ "አዎ" ከሆነ በሳምንት ምን ያህል ጊዜ ነዉ ጫት የሚቅሙት?	1. በየቀኑ<br>2. በሳምንት ከ5-6 ቀናት<br>3. በሳምንት ከ3-4 ቀናት<br>4. በሳምንት ከ1-2 ቀናት	
314	ለምን ያህል ጊዜ ጫት ቅመዋል?(በአመት ይገለጽ)	______አመት	
**ካፌን አጠቃቀም**			
315	ቡና ይጠጣሉ?	1. እጠጣለሁ<br>0. አልጠጣም	
316	ለጥያቄ 315 ምላሽዎ "አዎ" ከሆነ በሳምንት ስንት ቀን ቡና ይጠጣሉ?	1. በየቀኑ<br>2. በሳምንት ከ5-6 ቀናት<br>3. በሳምንት ከ3-4 ቀናት<br>4. በሳምንት ከ1-2 ቀናት	
317	በእነዚህ ቀናት ዉስጥ በቀን ስንት ሲኒ ቡና ይጠጣሉ?	1. በቀን አንድ ሲኒ<br>2. በቀን ሁለት ሲኒ<br>3. በቀን ሦስት ሲኒና በላይ	
**አመጋገብ**			
318	ፍራፍሬ በሳምንት ምን ያህል ቀናት ይመገባሉ?	_______ቀናት	
319	በእነዚህ ቀናት ውስጥ በቀን ስንት ጊዜ ፍራፍሬ ይመገባሉ? (ቁርስ ፣ምሳ፣ እራት፣ ወዘተ) ሲመገቡ	_______አቅርቦት ብዛት በቀን	
320	በሳምንት ውስጥ ምን ያህል ቀናት አትክልት ይመገባሉ?	_______ቀናት	
321	በእነዚህ ቀናት በቀን ስንት ጊዜ አትክልት ይመገባሉ? (ቁርስ ፣ምሳ፣ እራት፣ ወዘተ)	_______አቅርቦት ብዛት በቀን	

322	ቅቤና ጮማ የበዛበት ምግቦችን ይመገባሉ?	1. አዎ<br>0. አልመገብም	
323	ጮማ በሳምንት ለምን ያህል ቀናት ይመገባሉ?	_______ቀናት	
324	በእነዚህ ቀናት በቀን ምን ያህል ጊዜ ጮማ ያለበትን ምግብ የመገባሉ?<br>(ቁርስ ፣ምሳ እራት ወዘተ)	_______አቅርቦት ብዛት በቀን	
የጨው አጠቃቀም			
325	ጨዉ የተጨመረ ምግብ ይመገባሉ?	1. አዎ<br>0. አልመገብም	
326	በምግብ ውስጥ ከተጨመረው በላይ ተጨማሪ ጨው በገበታ ጨምረው ይመገባሉ?	1. አዎ<br>0. አልመገብም	
የሰውነት እንቅስቃሴ ሁኔታ			
327	በየዕለቱ የሚሰሩት ስራ ቢያንስ ያለማቋረጥ ለ10 ደቂቃ እንቅስቃሴ እና ጉልበት የሚጠይቅና አተነፋፈስንና የልብ ምት ፍጥነትን የሚጨምር ነው?	1. አዎ<br>0. አይደለም	
328	በመደበኛ ሳምንት ለስንት ቀን የጉልበት ስራ እንደመደበኛ ሥራዎት ይሰራሉ?	_______ቀናት	
329	በቀን ለምን ያህል ጊዜ በጉልበት ስራ ላይ ያሳልፋሉ?	_______ሰዓታት	
ከቦታ ቦታ መንቀሳቀስ			
330	በቀን ቢያንስ ያለማቋረጥ ለ10 ደቂቃ በእግርዎ ከቦታ ቦታ ይንቀሳቀሳሉ?	1. አዎ<br>0. አልንቀሳቀስም	
331	በመደበኛ ሳምንት ውስጥ ቢያንስ ያለማቋረጥ ለ10 ደቂቃ ያህል ምን ያህል ቀናት የእግር ጉዞ ያደርጋሉ?	_______ቀናት	
332	በአንድ ቀን ውስጥ ቢያንስ ምን ያህል ጊዜ በእግር ይጓዛሉ?	_______ሰዓታት<br><br>-------------ደቂቃ	
333	ከቦታ ቦታ ለመንቀሳቀስ የሚጠቀሙት መጓጓዣ ምንድን ነዉ?	1. እግር<br>2. ብስክሌት<br>3. ተሽከርካሪ	
የመዝናኛ እንቅስቃሴ			
334	በሚዝናኑበት ጊዜ ጉልበት የሚጠይቅ እና አተነፋፈስንና የልብ ምት ፍጥነትን በኃይል የሚጨምር የአካል ብቃት እንቅስቃሴ ወይም ስፖርት(ሩጫ፣እግር ኳስ…ወዘተ) ይጫወታሉ/ይሠራሉ?	1. አዎ<br>0. አልሰራም	
335	በሳምንት ውስጥ ስንት ቀን ጉልበት		

	የሚጠይቅ የአካል ብቃት እንቅስቃሴ ያደርጋሉ ወይም በስፖርት ይዝናናሉ?	_______ቀናት	
336	በመደበኛ ቀን ውስጥ ለምን ያህል ጊዜ የአካል ብቃት እንቅስቃሴ ያደርጋሉ ወይም ስፖርት በመሥራት ይዝናናሉ?	_______ደቂቃ	
**ክፍል አራት፡ ከደም ግፊት በሽታ ጋር የተያያዘ ታሪክ እና ተዛማጅ በሽታዎች**			
ቁጥር	ጥያቄ	ምላሽ	ምርመራ
401	የደም ግፊትዎን በሀኪም ወይም በሌላ የጤና ባለሙያ ተለክተው ያውቃሉ?	1. አዎ<br>0. አላውቅም	
402	የደም ግፊት በሽታ አለብዎት ተብለው በሀኪም ወይም በሌላ የጤና ባለሙያ ተነግሮት ያዉቃል?	1. አዎ<br>0. አላውቅም	
403	በአሁኑ ጊዜ በሀኪም ወይም በሌላ የጤና ባለሙያ የታዘዘልዎት ለደም ግፊት በሽታ መቆጣጠሪያ የሚወስዱት መድሀኒት ወይም ምክርና ህክምና አለ?	1. አዎ<br>0. የለም	
404	በቤተሰብዎ መካከል(አባት፣እናት፣ወንድምና እህት) የደም ግፊት በሽታ ያለበት ሰው አለ?	1. አዎ<br>0. አላውቅም	
405	የስኳር በሽታ አለብዎት ተብሎ በሀኪም ወይም በሌላ የጤና ባለሙያ ተነግሮት ያውቃል	1. አዎ<br>0. አላውቅም	

**ክፍል አምስት፡ የሰውነት ልኬት**			
**ቁመት እና ክብደት**			
ቁጥር	ልኬት	ዉጤት	ምርመራ
501	ቁመት	_________ሳ.ሜ.	
502	ክብደት	________ኪ.ግ.	
503	የዳሌ መጠነ ዙሪያ	_________ሳ.ሜ.	
504	የወገብ መጠነ ዙሪያ ልኬት	_________ሳ.ሜ.	
**የደም ግፊት ልኬት** በ5 ደቂቃ ልዩነት			
505	ንባብ 1	___ ሲስቶሊክ (ሚ.ሜ.ሜርኩሪ)	

		_____ዳያስቶሊክ (ሚ.ሜ.ሜርኩሪ)	
506	ንባብ 2	_____ ሲስቶሊክ(ሚ.ሜ.ሜርኩሪ)	
		_____ ዳያስቶሊክ (ሚ.ሜ.ሜርኩሪ)	
507	ንባብ 3	_____ ሲስቶሊክ (ሚ.ሜ.ሜርኩሪ)	
		_____ ዳያስቶሊክ (ሚ.ሜ.ሜርኩሪ)	
