# Supplementary material for: Prevalence and risk factors of hypertension among civil servants in Sidama Zone, south Ethiopia
Source: PLoS One. 2020 Jun 11;15(6):e0234485. doi: 10.1371/journal.pone.0234485 (PMC7289366; doi:10.1371/journal.pone.0234485)
Supplement: Attachment — Submitted filename: PONE-D-19-34743_Editor Comments.pdf [file pone.0234485.s004.pdf]

# PLOS ONE

## Prevalence and risk factors of hypertension among civil servants in Sidama Zone, south Ethiopia

--Manuscript Draft--

| | |
|---|---|
| **Manuscript Number:** | PONE-D-19-34743 |
| **Article Type:** | Research Article |
| **Full Title:** | Prevalence and risk factors of hypertension among civil servants in Sidama Zone, south Ethiopia |
| **Short Title:** | Hypertension among civil servants |
| **Corresponding Author:** | Bedilu Badego, MPH<br>Sidama Zone Health Department<br>Hawassa, Sidama Regional state ETHIOPIA |
| **Keywords:** | hypertension; Prevalence; blood pressure; risk factor; Ethiopia |
| **Abstract:** | Background: Hypertension is the leading cause of death and disability in adult populations globally. It is estimated that globally 1.13 billion people are hypertensive. More than two-third of the people with hypertension live in low- and middle-income countries. However, there is lack of data on the prevalence and risk factors of hypertension among civil servants working in various sectors and levels.<br>Objective: To assess the prevalence and risk factors of hypertension among civil servants in Sidama Zone, south Ethiopia.<br>Methods and Materials: An institution-based cross-sectional study was conducted from March 1-30, 2019 on a sample of 546 civil servants selected randomly from different departments of Sidama Zone Administration. Data were collected using structured, face-to-face interviewer-administered questionnaire and standard physical measurements. The data were entered using Epi Data 3.1 and analyzed using SPSS version 20. Multivariable logistic regression analysis was ~~done~~ to identify factors associated with hypertension. Adjusted odds ratios (AORs) with 95% confidence interval were computed to assess the presence and strength of associations.<br>Results: A total of 546 civil servants responded ~~with a~~ response rate of 94.9%. The prevalence of hypertension was 24.5% [95% confidence interval (CI): 23.3-25.6]. The identified risk factors ~~for~~ hypertension were male sex (AOR 4.31[95% CI: 1.84 – 10.09]), moderate current alcohol consumption (AOR: 4.85; [95% CI: 1.73- 13.61]), current khat chewing (AOR 2.97[95% CI: 1.38-6.40]), old age (AOR: 4.41[95% CI: 1.19 - 16.26]), being obese (AOR 5.94 [95% CI: 1.26 - 27.86]) and central obesity (AOR 3.57 [95% CI: 1.80 -7.07]).<br>Conclusions: One in four civil servants are hypertensive. Different demographic, behavioral and metabolic factors increase the ~~risk~~ of hypertension among civil servants. Local health authorities and concerned stake holders should work on awareness creation and health life style promotion among civil servants. |
| **Order of Authors:** | Bedilu Badego, MPH |
| | Amanuel Yoseph |
| | Ayalew Astatkie |
| **Opposed Reviewers:** | |
| **Additional Information:** | |
| **Question** | **Response** |
| **Financial Disclosure**<br><br>Enter a financial disclosure statement that describes the sources of funding for the work included in this submission. Review the submission guidelines for detailed requirements. View published research | The author(s) received no specific funding for this work. |

Powered by Editorial Manager® and ProduXion Manager® from Aries Systems Corporation

| | |
|---|---|
| articles from *PLOS ONE* for specific examples.<br><br>This statement is required for submission and **will appear in the published article** if the submission is accepted. Please make sure it is accurate.<br><br>**Unfunded studies**<br>Enter: *The author(s) received no specific funding for this work.*<br><br>**Funded studies**<br>Enter a statement with the following details:<br>• Initials of the authors who received each award<br>• Grant numbers awarded to each author<br>• The full name of each funder<br>• URL of each funder website<br>• Did the sponsors or funders play any role in the study design, data collection and analysis, decision to publish, or preparation of the manuscript?<br>• **NO** - Include this sentence at the end of your statement: *The funders had no role in study design, data collection and analysis, decision to publish, or preparation of the manuscript.*<br>• **YES** - Specify the role(s) played.<br><br>* typeset | |
| **Competing Interests**<br><br>Use the instructions below to enter a competing interest statement for this submission. On behalf of all authors, disclose any competing interests that could be perceived to bias this work—acknowledging all financial support and any other relevant financial or non-financial competing interests.<br><br>This statement **will appear in the published article** if the submission is accepted. Please make sure it is accurate. View published research articles from *PLOS ONE* for specific examples. | The authors have declared that no competing interests exist. |

Powered by Editorial Manager® and ProduXion Manager® from Aries Systems Corporation

| | |
|---|---|
| **NO authors have competing interests**<br><br>Enter: *The authors have declared that no competing interests exist.*<br><br>**Authors with competing interests**<br><br>Enter competing interest details beginning with this statement:<br><br>*I have read the journal's policy and the authors of this manuscript have the following competing interests: [insert competing interests here]*<br><br>* typeset | |
| **Ethics Statement**<br><br>Enter an ethics statement for this submission. This statement is required if the study involved:<br><br>• Human participants<br>• Human specimens or tissue<br>• Vertebrate animals or cephalopods<br>• Vertebrate embryos or tissues<br>• Field research<br><br>Write "N/A" if the submission does not require an ethics statement.<br><br>General guidance is provided below. Consult the submission guidelines for detailed instructions. **Make sure that all information entered here is included in the Methods section of the manuscript.** | Ethical clearance was obtained from the Institutional Review Board (IRB) at the College of Medicine and Health Sciences of Hawassa University before commencing data collection (Ref. No: IRB/041/11). A letter of support was obtained from Sidama Zone Administration and administrative permissions was obtained from every department where the study was conducted. The purposes of the study and the participant’s full right whether or not to participate in the study based on only their volunteerism was explained in detail to the study participants. Privacy and confidentiality of the study participants were assured maximally. Study participants with high blood pressure were referred to nearby health facilities for further diagnosis and treatment. |

Powered by Editorial Manager® and ProduXion Manager® from Aries Systems Corporation

| | |
|---|---|
| **Format for specific study types**<br><br>**Human Subject Research (involving human participants and/or tissue)**<br>• Give the name of the institutional review board or ethics committee that approved the study<br>• Include the approval number and/or a statement indicating approval of this research<br>• Indicate the form of consent obtained (written/oral) or the reason that consent was not obtained (e.g. the data were analyzed anonymously)<br><br>**Animal Research (involving vertebrate animals, embryos or tissues)**<br>• Provide the name of the Institutional Animal Care and Use Committee (IACUC) or other relevant ethics board that reviewed the study protocol, and indicate whether they approved this research or granted a formal waiver of ethical approval<br>• Include an approval number if one was obtained<br>• If the study involved *non-human primates*, add *additional details* about animal welfare and steps taken to ameliorate suffering<br>• If anesthesia, euthanasia, or any kind of animal sacrifice is part of the study, include briefly which substances and/or methods were applied<br><br>**Field Research**<br><br>Include the following details if this study involves the collection of plant, animal, or other materials from a natural setting:<br>• Field permit number<br>• Name of the institution or relevant body that granted permission | |
| **Data Availability**<br><br>Authors are required to make all data underlying the findings described fully available, without restriction, and from the time of publication. PLOS allows rare exceptions to address legal and ethical concerns. See the PLOS Data Policy and FAQ for detailed information. | Yes - all data are fully available without restriction |

Powered by Editorial Manager® and ProduXion Manager® from Aries Systems Corporation

| | |
|---|---|
| A Data Availability Statement describing where the data can be found is required at submission. Your answers to this question constitute the Data Availability Statement and **will be published in the article**, if accepted.<br><br>**Important:** Stating 'data available on request from the author' is not sufficient. If your data are only available upon request, select 'No' for the first question and explain your exceptional situation in the text box.<br><br>Do the authors confirm that all data underlying the findings described in their manuscript are fully available without restriction? | |
| **Describe where the data may be found in full sentences. If you are copying our sample text, replace any instances of XXX with the appropriate details.**<br><br>• If the data are **held or will be held in a public repository**, include URLs, accession numbers or DOIs. If this information will only be available after acceptance, indicate this by ticking the box below. For example: *All XXX files are available from the XXX database (accession number(s) XXX, XXX.*).<br>• If the data are all contained **within the manuscript and/or Supporting Information files**, enter the following: *All relevant data are within the manuscript and its Supporting Information files.*<br>• If neither of these applies but you are able to provide **details of access elsewhere**, with or without limitations, please do so. For example:<br><br>*Data cannot be shared publicly because of [XXX]. Data are available from the XXX Institutional Data Access / Ethics Committee (contact via XXX) for researchers who meet the criteria for access to confidential data.*<br><br>*The data underlying the results presented in the study are available from (include the name of the third party* | The authors will provide the data underlying this study if the manuscript is accepted for publication. |

| | |
|---|---|
| *and contact information or URL).*<br>• This text is appropriate if the data are owned by a third party and authors do not have permission to share the data.<br><br>* typeset | |
| Additional data availability information: | |

Powered by Editorial Manager® and ProduXion Manager® from Aries Systems Corporation

# Cover letter

Dear Editor-in-Chief,

*PLOS ONE*

We are submitting a manuscript of an original research on prevalence and risk factors of hypertension among civil servants in Ethiopia to be considered for publication by your esteemed journal, to *PLOS ONE*. The current study specifically addresses the prevalence and risk factors of hypertension among civil servants, in an administrative zone in southern Ethiopia. With the rise of chronic non-commucable diseases, particularly hypertension, in less developed countries, the study highlights the magnitude and possible risk factors for hypertension among civil servants. The findings of the present study would help in context-specific intervention mechanisms to reduce the epidemic of hypertension among civil servants. We therefore believe that the present research falls within the scope of *PLOS ONE*.

We testify that this manuscript has not been published or is not under consideration for publication elsewhere.

With regards,

Bedilu Badego (baynbadego@gmail.com)

Amanuel Yoseph (amanuelyoseph45@gmail.com)

Ayalew Astatkie (*ayalewastatkie@gmail.com*)

Manuscript Click here to download Manuscript Manuscript.docx

**RESEARCH ARTICLE**

# Prevalence and risk factors of hypertension among civil servants in Sidama Zone, south Ethiopia

**Bedilu Badego [1*], Amanuel Yoseph [2], Ayalew Astatkie [2]**

**[*]Corresponding author**

**Authors' address**

[1] **Bedilu Badego: Sidama Zone Health Department, Hawassa, Ethiopia, P.O.Box 52; Tel: +251911920697; E-mail:** baynbadego@gmail.com

[2] **Amanuel Yoseph: School of Public Health, College of Medicine and Health Sciences, Hawassa University, Hawassa, Ethiopia. P. Box 05; Tel: +251915683578; E-mail:** amanuelyoseph45@gmail.com

[2] **Ayalew Astatkie: School of Public Health, College of Medicine and Health Sciences, Hawassa University, Hawassa, Ethiopia. P. Box 05; Tel: +251911739694; E-mail:** ayalewastatkie@gmail.com

## Abstract

**Background:** Hypertension is the leading cause of death and disability in adult populations globally. It is estimated that globally 1.13 billion people are hypertensive. More than two-third of the people with hypertension live in low- and middle-income countries. However, there is lack of data on the prevalence and risk factors of hypertension among civil servants working in various sectors and levels.

**Objective:** To assess the prevalence and risk factors of hypertension among civil servants in Sidama Zone, south Ethiopia.

**Methods and Materials:** An institution-based cross-sectional study was conducted from March 1-30, 2019 on a sample of 546 civil servants selected randomly from different departments of Sidama Zone Administration. Data were collected using structured, face-to-face interviewer-administered questionnaire and standard physical measurements. The data were entered using Epi Data 3.1 and analyzed using SPSS version 20. Multivariable logistic regression analysis was done to identify factors associated with hypertension. Adjusted odds ratios (AORs) with 95% confidence interval were computed to assess the presence and strength of associations.

**Results:** A total of 546 civil servants responded with a response rate of 94.9%. The prevalence of hypertension was 24.5% [95% confidence interval (CI): 23.3-25.6]. The identified risk factors for hypertension were male sex (AOR 4.31[95% CI: 1.84 – 10.09]), moderate current alcohol consumption (AOR: 4.85; [95% CI: 1.73- 13.61]), current khat chewing (AOR 2.97[95% CI: 1.38-6.40]), old age (AOR: 4.41[95% CI: 1.19 - 16.26]), being obese (AOR 5.94 [95% CI: 1.26 - 27.86]) and central obesity (AOR 3.57 [95% CI: 1.80 -7.07]).

**Conclusions:** One in four civil servants are hypertensive. Different demographic, behavioral and metabolic factors increase the risk of hypertension among civil servants. Local health authorities and concerned stake holders should work on awareness creation and health life style promotion among civil servants.

# Introduction

Non-communicable diseases (NCD) are the major cause of morbidity and premature deaths under the age of 70 years globally. Nearly half of NCD deaths occur due to cardiovascular diseases (CVD). Elevated blood pressure is a major risk factor for cardiovascular diseases [1].

According to the Global Burden of Disease Study report, hypertension is the leading cause for death and disability among adults globally [2]. In adults older than 25 years, approximately 4 in 10 individuals are hypertensive and nowadays hypertension is a public health epidemic across the world [3]. Hypertension is also a leading risk for dementia, renal failure, and fetal and maternal death in pregnancy [4, 5].

It is estimated that globally 1.13 billion people are hypertensive and the overall prevalence of hypertension in adults is around 30 - 45% with 10 million deaths and above 200 million disability-adjusted life years (DALYs), in 2015 [6, 7].

This high prevalence of raised blood pressure is consistent across the world, irrespective of socio-economic status, i.e. in lower, middle, and higher income countries. However, nearly four-fifth of deaths due to CVDs occur in low-and middle-income countries (LMICs) [8].

The burden of hypertension disproportionately impacts low-and middle-income countries. ~~Heart disease and stroke occur in younger people and two-thirds of people with hypertension live in economically developing countries [3].~~ Diseases related to elevated blood pressure have a greater impact on healthcare expenditure, and raised blood pressure and its complications cost an estimated 10% of healthcare spending [9].

Recent studies conducted in Ethiopia showed that the prevalence of CVD risk factors increased rapidly [10]. In Ethiopia nationally representative survey on prevalence and risk factors of

hypertension is not available. However, a number of small-scale studies have reinforced the estimate. The prevalence of raised blood pressure in Ethiopia ranges from 19.6% to 30.2% [11, 12].

In general, in lower-and middle-income countries including Ethiopia, the burden of diseases has been shifting to non-communicable diseases from communicable diseases due to unplanned rapid urbanization of rural communities and life style changes in populations in terms of nutrition, physical inactivity, and increase in behaviors such as harmful use of alcohol, tobacco use and drug use in both rural and urban residents. Therefore, prevention, early detection and control program for hypertension is very important to reduce the impact of its burden on health and economy [13].

Besides, prevention and control of hypertension in Ethiopia has not received due attention due to limited health care budget, so that the health care system gives high priority for prevention and control of communicable diseases like tuberculosis, HIV/AIDS, malaria and maternal health [14]. Moreover, while civil servants are likely to be at an increased risk for hypertension due to sedentary lifestyle, stress and other factors that are peculiar to civil servants, there is limited information on prevalence and risk factors of hypertension among civil servants in Ethiopia, so much so in the Sidama Zone of southern Ethiopia. Therefore, this study was conducted to determine prevalence and risk factors of hypertension among civil servants in different departments of Sidama Zone, southern Ethiopia.

# Methods and Materials

## Study setting

The study was conducted in Sidama zone, south Ethiopia. Sidama zone is one of the eighteen zones and three special districts in the Southern Nations Nationalities and Peoples Region and located 275 km south of Addis Ababa. It consists 30 districts and 6 town administrations with a total of 576 kebeles. Based on the Ethiopian Population Census Report 2007 projections, the total population of the Zone in 2019 has reached 3,893,816.

The Zone administration has a total of 4806 health professionals of different disciplines and 524 health posts, 125 health centers, one general hospital and 13 primary hospitals owned by the government. There are also 21 private medium and three nongovernmental (NGO) clinics, and 63 private drug stores. The overall potential health service coverage of the zone by public health facilities is 90.3%.

Hawassa city is the capital city of the Sidama Zone Administration. Currently there are 26 zonal departments/ offices administered under Sidama Zone Administration and all are located in the capital, Hawassa city. Each department/ office has its own distinct responsibilities for serving the community.

## Study design and population

An institution-based cross-sectional study design was conducted from March 1-30, 2019 among civil servants of Sidama Zone. The source population for the study comprised of all civil servants who were working in different departments of Sidama Zone Administration while the study

population constituted randomly selected civil servants. Pregnant civil servants were excluded from the study, because of preeclampsia or pregnancy induced hypertension.

### Sample Size Determination

The sample size was calculated using Epi-Info™ version 7 with prevalence of hypertension 37.7% taken from Akaki Steel Factory [15], margin of error (d) 0.05, 95% confidence level 1.96 and a 10% non-response rate. Accordingly, the final sample size was 402.

The sample size for the second objective was also calculated by considering variables significantly associated with hypertension in studies conducted Akakai Steel Factory, [15], Northwest Ethiopia [16] and federal ministries of Ethiopia [17]. The sample sizes ~~accordingly~~ calculated were 263, 575 and 283. Hence the sample size of 575 was used as the final sample size for the present study as it would suffice to address all objectives of the study.

## Sampling procedure

The study participants were selected from the complete list of all civil servants which was obtained from Sidama Zone Finance and Economic Development Department's payroll. The list on the payroll served as a sampling frame.

Sidama Zone Administration comprises 26 departments/ zonal offices with a total of 1,341 civil servants, who were on duty during November 2018 [Personal Communication, Sidama Zone Finance and Economic Development Department, 2018]. All departments were included in the study and the total sample size was apportioned for each department proportional to the size of the number of employees of the respective departments. For departments which had small number of employees and, as a result, for which the allocated sample sizes were very small, there was over-sampling to ensure a better representation of the workers in such departments.

Therefore, all departments with sample size less than 15 were boosted to 15 and departments with sample sizes greater than 35 were downsized to 35. Then, civil servants were stratified into three categories based on their responsibility as higher level or executives, technical workers or experts and supportive staffs (drivers, secretaries, guards, janitors, etc.) in order to give sufficient representation for all employees to participate in the study. The sample size allocated to a given department was also be proportionally allocated to the three strata within a department.

Proportionally allocated study subjects were selected using simple random sampling method from each stratum or category. All individuals were selected by simple random sampling method until the intended sample size was reached.

When randomly selected employees were absent during data collection time, the interview was conducted in the second or third visit by communicating with them the convenient time within the data collection period. But, study subjects who were absent in the third visit for different reason or unwilling to participate in the study were considered non-respondents.

## Data collection tools and procedures

Data were collected using well structured, face-to-face interviewer-administered questionnaire and standard physical measurements adapted from "The WHO STEP wise approach to chronic disease risk factor surveillance (STEPS)" [18]. The questionnaire was prepared in English language and translated to local (Amharic) language and translated back to English to check for consistency.

Physical measurements included weight, height, waist circumference, hip circumference and blood pressure measurement but did not include biochemical measurements due to resource constraints. Standard functional mercury sphygmomanometers and stethoscope were used to

measure the blood pressure with calibration or adjustment to zero level for each measurement and participant. Before measuring blood pressure, the participant was asked to rest five to ten minutes in a sitting position with legs uncrossed. They were also asked to confirm that they had not smoked or consumed caffeine-containing products for at least 30 minutes prior to measurements. If participants had smoked or used caffeine within 30 minutes prior to BP measurement, data collectors told them with justification to stay there at least for 30 minutes. Three consecutive measurements were taken in an interval of at least five minutes. Mean systolic and diastolic blood pressures were determined by averaging the second and third measurements [3, 19].

Body weight was measured using standardized adult weighing scale with the subjects standing, arms hanging naturally at the sides, wearing light clothing and without footwear. Similar weighing scale was used for all subjects, calibrated for each individual and the reading is expressed in kilograms (kg) to the nearest 0.5 kg [20]. Height of the study subjects was measured in meters using a stadio-meter, with the subjects standing upright, facing straight forward, barefooted, and measurement was expressed to the nearest centimeter [21].

Six nurses with a bachelor's degree and two health officers were recruited from public health institutions of the study area for data collection and supervision, respectively. Then, data collectors and supervisors were trained for two days on the objectives of the study, data collection tools, interview techniques, and their roles and responsibilities by the principal investigator. At the end of the training, the questionnaire was pre-tested on 5% of a total sample size on civil servants in Hawassa City Administration before the actual data collection. Regular supervision of data collectors ~~for checking~~ completeness and accuracy of the data were made by supervisors and the principal investigator.

## Study variable

The outcome variable was hypertension status which was defined as a systolic blood pressure equal to or above 140 mm Hg and/or diastolic blood pressure equal to or above 90 mm Hg, or any prior diagnosis of hypertension made by a health professional, and taking antihypertensive drugs [3]. Independent variables were: socio-demographic variables such as age, sex, marital status, religion, family size, monthly income, occupations, educational status, and residence; behavioral or lifestyle factors like cigarette smoking, alcohol drinking, physical inactivity, unhealthy diets, additional salt use, khat (*Catha edulis*) chewing, and coffee drinking; disease history such as personal history of diabetes mellitus, and family history of hypertension; and metabolic factors like overweight, obesity, central obesity, diabetes mellitus.

## Data Analysis procedure

The data were edited, coded and entered to Epi Data 3.1 and exported to the Statistical Package for Social Sciences (SPSS) version 20.0 for analysis. Descriptive analyses were conducted to describe the socio-demographic characteristics and other variables. Adjusted odds ratios from multivariable logistic regression were used to measure presence and strength of association between hypertension and independent variables. Variables with a p-value <0.25 in bivarible analysis were candidates for the multivariable logistic regression. The goodness- of- fit was tested by Hosmer-Lemeshow statistic (p-value >0.05). Multicollinearity between independent variables was also assessed but no evidence of multicollinearity was found.

### Ethics Statement

Ethical clearance was obtained from the Institutional Review Board (IRB) at the College of Medicine and Health Sciences of Hawassa University before commencing data collection (Ref.

No: IRB/041/11). A letter of support was obtained from Sidama Zone Administration and administrative permissions was obtained from every department where the study was conducted. The purposes of the study and the participant's full right whether or not to participate in the study based on only their volunteerism was explained in detail to the study participants. Privacy and confidentiality of the study participants were assured maximally. Study participants with high blood pressure were referred to nearby health facilities for further diagnosis and treatment.

# Results

## Socio-demographic characteristics of the study participants

Out of 575 eligible study participants, a total of 546 civil servants took part in the study with a response rate of 94.9%. Thirteen (2.2%) study participants were not around because of annual leave and 16 (2.8%) civil servants were not willing to participate in the study for different reasons. Among the total respondents 356 (65.2%) were males and the mean (+standard deviation) of the age of participants was 37.86 (+9.50) years. See details of the socio-demographic characteristics of the study participants in Table 1.

Table 1: Socio-demographic characteristics of the study participants in Sidama zone, South Ethiopia, 2019.

| **Variable (n=546)** | **Categories** | **Frequency** | **Percent** |
|---|---|---|---|
| Sex | Male | 356 | 65.2 |
| | Female | 190 | 34.8 |
| Age | 18-29 | 119 | 21.8 |
| | 30-39 | 194 | 35.5 |
| | 40-49 | 149 | 27.3 |
| | ≥50 | 84 | 15.4 |
| Marital status | Single | 77 | 14.1 |
| | Married | 410 | 75.1 |

| | | | |
|---|---|---|---|
| | Divorced | 32 | 5.9 |
| | Widowed | 27 | 4.9 |
| Educational status | Primary education 1-8 | 8 | 1.5 |
| | Secondary education 9-12 | 45 | 8.2 |
| | Diploma and above | 493 | 90.3 |
| Task level | High level managerial work | 125 | 22.9 |
| | Experts | 318 | 58.2 |
| | Supportive staffs | 103 | 18.9 |
| Family size | 1-5 | 304 | 55.7 |
| | >5 | 242 | 44.3 |
| Monthly income quintile (birr) | Lowest | 98 | 17.9 |
| | Second lowest | 102 | 18.7 |
| | Middle | 148 | 27.1 |
| | Second highest | 97 | 17.8 |
| | Highest | 97 | 17.8 |

### History of substance use

From the 546 study participants, 76 (13.9%) had smoked cigarettes at least once in their lifetime, and only two (0.4%) were currently smoking (in the past four weeks). The two currently smoking participants were smoking daily for more than 20 years as light smokers. Two hundred nineteen (40.1%) of the respondents were past drinkers, while 90 (16.5%) were still drinking.

Concerning khat chewing, 138 (25.3%) participants reported chewing khat at least once in their life time, whereas 69 (12.6%) were current chewers. Further, 515 (94.3%) study participants were coffee drinkers, of whom 479 (93%) were drinking coffee on a daily basis (Table 2).

Table 2: History of substance use of the study participants in Sidama zone, south Ethiopia, 2019.

| **Variable (n=546)** | **Categories** | **Frequency** | **Percent** |
|---|---|---|---|
| Ever smoked cigarette | Yes | 76 | 13.9 |
| | No | 470 | 86.1 |
| Currently cigarette smoking | Yes | 2 | 0.4 |
| | No | 544 | 99.6 |
| Ever drunk alcohol | Yes | 219 | 40.1 |
| | No | 327 | 59.9 |
| Currently alcohol drinking | Yes | 90 | 16.5 |
| | No | 456 | 83.5 |

| | | | |
|---|---|---|---|
| Level of current standard alcohol drink (n=90) | Low | 51 | 56.6 |
| | Moderate | 39 | 43.4 |
| | No | 457 | 83.7 |
| Ever chewed khat | Yes | 138 | 25.3 |
| | No | 408 | 74.7 |
| Currently khat chewing | Yes | 69 | 12.6 |
| | No | 447 | 87.4 |
| Khat chewing days per week (n=69) | 5 days and more | 4 | 5.8 |
| | 3-4 days | 28 | 40.6 |
| | 1-2 days | 37 | 53.6 |
| Coffee drinking | Yes | 515 | 94.3 |
| | No | 31 | 5.7 |
| Days of coffee drinking per week (n=515) | Daily | 479 | 93 |
| | 5-6 days | 7 | 1.4 |

| | | | |
|---|---|---|---|
| | 3-4 | 9 | 1.7 |
| | 1-2 | 20 | 3.9 |
| Cups of coffee consumed per day (n=515) | 1 cup | 86 | 16.7 |
| | 2 cups | 149 | 28.9 |
| | 3 and more cups | 280 | 54.4 |

**Physical activity of the study participants**

Among the study participants 173 (31.7%) reported they were involved in work related vigorous activities, of whom 94 (54.3%) performed 4-7 days per week. The rest performed 1-3 days per week. Majority (78%) of participants had one hour of work-related vigorous activity per day. About 90% of all respondents reported they practiced brisk walking at least 10 minutes per day for 1-7 days in a week, and 55.8% practiced it on daily basis.

From the study participants, 116 (21.2%) were practicing sports and recreational vigorous physical activities, of whom 43% practiced one to two days per week. Overall, 385 (70.5%) of the study participants were physically active in one or more categories of physical activities (Table 3).

Table 3: Physical activities of the study participants in Sidama zone, south Ethiopia, 2019.

| **Variable (n=546)** | **Categories** | **Frequency** | **Percent** |
|---|---|---|---|

| | | | |
|---|---|---|---|
| Work related vigorous activity | Yes | 173 | 31.7 |
| | No | 373 | 68.3 |
| Vigorous work related activity days per week | 1-3 | 79 | 45.7 |
| | 4-7 | 94 | 54.3 |
| Vigorous work related activity hours per days | One hours | 135 | 78 |
| | Two hours | 28 | 16 |
| | 3-4 hours | 10 | 6 |
| Brisk walking at least for 10 minutes per day | Yes | 496 | 90.8 |
| | No | 50 | 9.2 |
| Frequency of brisk walking days per week | Daily | 277 | 55.8 |
| | 5-6 | 82 | 16.5 |
| | 3-4 | 90 | 18.2 |
| | 1-2 | 47 | 9.5 |
| Means of transportation | On foot | 17 | 3.1 |
| | Bicycle | 4 | 0.1 |
| | Vehicle with engine | 525 | 96.2 |

| | | | |
|---|---|---|---|
| Vigorous-intensity sports or recreational activity | Yes | 116 | 21.2 |
| | No | 430 | 78.8 |
| Frequency of vigorous intensity sport activity per week (n=116) | Daily | 9 | 7.8 |
| | 5-6 | 12 | 10.3 |
| | 4-3 | 45 | 38.8 |
| | 2-1 | 50 | 43.1 |
| Time spent for vigorous sport activity in minutes per day (n=116) | 10-30 | 62 | 53.4 |
| | >30 | 54 | 46.6 |
| Physical activity status | Active | 385 | 70.5 |
| | Inactive | 161 | 29.5 |

**Dietary practice of the study participants**

Among the participants 511 (93.6%) had the habit of consuming fruits and 538 (98.5%) had the habit of vegetable consumption; however, 45 (8.8%) were eating fruits and 176 (32.7%) were eating vegetables on daily basis as per the recommendation of WHO. Eighty six (15.8%) were using fatty foods of animal origin and 50 (9.2%) were using additional salt in their diet (Table 4).

Table 4: Dietary practice of studied participants in different department of Sidama zone, 2019.

| **Variable (n=546)** | **Categories** | **Frequency** | **Percent** |
|---|---|---|---|
| Fruit consumption | Yes | 511 | 93.6 |
| | No | 35 | 6.4 |
| Fruit consumption days per week (n=511) | ≤ 6 days | 466 | 91.2 |
| | Daily | 45 | 8.8 |
| Vegetable consumption | Yes | 538 | 98.5 |
| | No | 8 | 1.5 |
| Vegetable consumption days per week (n=538) | ≤ 6 days | 362 | 67.3 |
| | Daily | 176 | 32.7 |
| Use of animal fatty food | Yes | 86 | 15.8 |
| | No | 460 | 84.2 |
| Use additional salt | Yes | 50 | 9.2 |
| | No | 496 | 90.8 |

### Anthropometric characteristics of the study participants

Regarding the body mass index (BMI), 233 (42.7%) were overweight and 97 (17.8%) were obese. Concerning the central obesity, 275 (50.4%) of the screened participants had high waist-to-hip ratio (WHR $\geq$ 0.95 for males and $\geq$ 0.85 for females). The mean ($\pm$SD) BMI of the study participants were 26.2 ($\pm$ 4.0) and mean ($\pm$SD) WHR were 0.91 ($\pm$ 0.08) (Table 5).

Table 5: Anthropometric measurements of the study participants in Sidama zone, south Ethiopia, 2019.

| **Variable (n=546)** | **Categories** | **Frequency** | **Percent** |
|---|---|---|---|
| BMI category | Underweight | 12 | 2.2 |
| | Normal | 204 | 37.4 |
| | Overweight | 233 | 42.7 |
| | Obese | 97 | 17.8 |
| Mean BMI | | 26.2$\pm$ 4.0 | |
| WHR | High | 275 | 50.4 |
| | Low | 271 | 49.6 |

| | |
|---|---|
| Mean WHR | 0.91 ± 0.08 |

Note: BMI, body mass index; WHR, waist-to-hip ratio

**Prevalence of hypertension**

The prevalence of hypertension among civil servants in all departments of Sidama Zone Administration was 24.5% (95% CI: 23.3-25.6). The mean (±SD) systolic BP was 120.22 (± 15.03) mmHg and the mean (±SD) diastolic BP was 80.04 ± 9.51 mmHg. Majority of hypertension cases were males 102 (76.1%) and 32 (23.9%) were females.

Four hundred twenty nine (78.6%) participants had been ever measured their blood pressure. Among 134 hypertensive cases, 51 (38%) were aware of being hypertensive prior to the study, and of whom 24 (47%) were using anti-hypertensive medication; however, 8 (33.3%) of those on medication had controlled their blood pressure by medication.

Among screened participants, 83 (15.2%) were newly screened hypertensive cases and all of them were not aware of being hypertensive (Table 6)

Table 6: Prevalence of hypertension among study participants in Sidama Zone, south Ethiopia, 2019.

| **Variable (n=546)** | **Categories** | **Frequency** | **Percent** |
|---|---|---|---|
| Hypertension | Yes | 134 | 24.5 |
| | No | 412 | 75.5 |

| | | | |
|---|---|---|---|
| Ever been measured BP prior to screening | Yes | 429 | 78.6 |
| | No | 117 | 21.4 |
| Known hypertensive cases (n=134) | Yes | 51 | 38 |
| | No | 83 | 62 |
| Hypertension cases on medication (n=51) | Yes | 24 | 47 |
| | No | 27 | 53 |
| Newly screened hypertension positive | Yes | 83 | 15.2 |
| | No | 463 | 84.8 |
| HTN by systolic and diastolic (n=134) | Yes | 61 | 45.5 |
| | No | 73 | 54.4 |
| Family history of hypertension | Yes | 191 | 35 |
| | No | 355 | 65 |
| Controlled hypertension (n=24) | Yes | 8 | 33.3 |
| | No | 16 | 66.7 |
| Personal history of diabetes mellitus | Yes | 31 | 5.7 |
| | No | 515 | 94.3 |

| | | | |
|---|---|---|---|
| Level of blood pressure (n=522) | Normal | 322 | 61.7 |
| | Pre-hypertension | 90 | 17.2 |
| | Stage I | 79 | 15.1 |
| | Stage II | 31 | 5.9 |

### Risk factors for hypertension among civil servants

Binary logistic regression was carried out to determine the factors associated with hypertension. Age, sex, educational status, family's monthly income, marital status, family size, work task level, harmful use of alcohol, tobacco use, physical activity status, means of transportation, eating animal fat, fruit and vegetable consumption, use of additional salt in food, khat use, coffee drinking, having diabetes mellitus, family history of hypertension, waist-to-hip ratio, BMI and level of knowledge were considered in the bi-variable analyses. As revealed by bi-variable analysis age, sex, marital status, family size, family's monthly income, harmful use of alcohol, khat use, eating animal fat, using additional salt in meals, having diabetes mellitus, BMI and central obesity were found to be a candidate variables for the multivariable model ($p<0.25$).In the multivariable model, male sex, old age, current moderate alcohol drinking, current status of chewing khat, being obese and central obesity were found to be significantly associated with hypertension ($p<0.05$).

It was revealed that hypertension was more likely to occur in males as compared to female study participants (AOR: 4.31; 95% CI: 1.84 – 10.09). The odds of being diseased by hypertension increased 2.97 times for study participants who had a habit of current khat chewing (AOR: 2.97,

95% CI: 1.38-6.40) than study participants who did not chew khat. Moreover, being in old age (AOR: 4.41; 95% CI: 1.19 -16.26), having moderate level of current alcohol consumption (AOR: 4.85; 95% CI: 1.73- 13.61) and being obese (AOR: 5.94; 95% CI: 1.26-27.86) were identified as the risk factors for hypertension. Finally, multivariable analysis revealed that hypertension was more likely to occur in study participants who had central obesity as compared to those having no central obesity (AOR: 3.57; 95% CI: 1.80-7.07) (Table 7).

Table 7: Risk factors for hypertension among civil servants in different departments of Sidama zone, south Ethiopia, 2019.

| **Variable (n=546)** | **Hypertension status** | | **COR of 95% CI** | **AOR of 95% CI** |
|---|---|---|---|---|
| | **Yes (%)** | **No (%)** | | |
| Sex | | | | |
| Male | 102 (28.7) | 254 (71.3) | 1.98(1.27,3.09) | 4.31(1.84,10.09)** |
| Female | 32 (16.8) | 158 (83.2) | 1 | 1 |
| Age in years | | | | |
| 18-29 | 10 (8.4) | 109 (91.6) | 1 | 1 |
| 30-39 | 36 (18.6) | 158 (81.4) | 2.48(1.18,5.21) | 1.96(0.65,5.87) |
| 40-49 | 52 (34.9) | 97 (65.1) | 5.84(2.81,12.12) | 2.14(0.65,7.03) |
| ≥50 | 48 (42.9) | 36 (57.1) | 8.17(3.75,17.80) | 4.41(1.19,16.26)* |

| | | | | |
|---|---|---|---|---|
| Marital status | | | | |
| Single | 10 (13.0) | 67 (87.0) | 1 | 1 |
| Married | 109 (26.6) | 301 (73.4) | 2.42(1.20,4.88) | 1.15(0.37,3.55) |
| Divorced | 8 (25.0) | 24 (75.0) | 2.23(0.78,6.31) | 1.24(0.25,5.96) |
| Widowed | 7 (25.9) | 20 (74.1) | 2.34(0.79,6.95) | 2.40(0.42,13.61) |
| Family size | | | | |
| 1-5 | 60 (19.7) | 244 (80.3) | 1 | 1 |
| >5 | 74 (30.6) | 168 (69.4) | 1.79(1.20,2.54) | 0.60(0.29,1.25) |
| Family monthly Income (n=542) | | | | |
| Lowest | 17 (17.3) | 81 (82.7) | 1 | 1 |
| Second lowest | 25 (24.5) | 77 (75.5) | 1.54(0.77, 3.08) | 0.54(0.19,1.49) |
| Middle | 45 (30.4) | 103 (69.6) | 2.08(1.10, 3.90) | 0.87(0.34,2.24) |
| Second highest | 25 (25.8) | 72 (74.2) | 1.65(0.82, 3.30) | 0.92(0.30,2.75) |
| Highest | 22 (22.7) | 75 (77.3) | 1.39(0.69, 2.83) | 0.60(0.20,1.76) |
| Current level of | | | | |

| | | | | |
|---|---|---|---|---|
| alcohol use | | | | |
| No | 93 (20.4) | 363 (79.6) | 1 | 1 |
| Low | 19 (37.3) | 32 (62.7) | 2.31(1.25,4.27) | 1.57(0.60,4.15) |
| Moderate | 22 (56.4) | 17 (43.6) | 5.05(2.57,9.89) | 4.85(1.73,13.61)** |
| Current khat Chewing | | | | |
| Yes | 71 (50.7) | 67 (49.3) | 3.93(2.33, 6.61) | 2.97(1.38,6.40)** |
| No | 63 (20.8) | 345 (79.2) | 1 | 1 |
| Eating animal fat | | | | |
| Yes | 28 (32.6) | 58 (67.4) | 1.61(0.97, 2.65) | 2.12(0.95,4.71) |
| No | 106 (23.0) | 354 (77.0) | 1 | 1 |
| Use additional salt | | | | |
| Yes | 8 (16.0) | 42 (84.0) | 1.78(0.81, 3.91) | 0.76(0.25, 2.28) |
| No | 126 (25.4) | 370 (74.6) | 1 | 1 |
| Having Diabetes Mellitus | | | | |

| | | | | | |
|---|---|---|---|---|---|
| | Yes | 15 (48.4) | 16 (51.6) | 3.12(1.49,6.49) | 1.42(0.44, 4.58) |
| | N o | 119 (23.1) | 396 (76.9) | 1 | 1 |
| BMI | | | | | |
| | <18.5 | 5 (41.7) | 7 (58.3) | 1 | 1 |
| | 18.5-24.99 | 17 (8.3) | 187 (91.7) | 1.56(0.19, 12.62) | 0.07(0.01, 0.31) |
| | 25-29.99 | 30 (12.9) | 203 (87.1) | 2.94(0.37, 13.42) | 0.08(0.01, 0.32) |
| | ≥30 | 82 (84.5) | 15 (15.5) | 12.15(3.52, 27.1) | 5.94(1.26,27.86)** |
| Central obesity | | | | | |
| | Yes | 100 (36.4) | 175 (63.6) | 3.98(2.57, 6.15) | 3.57(1.80, 7.07)** |
| | No | 34 (12.5) | 237 (87.5) | 1 | 1 |

**Note:**1: Indicates the reference categories; *: Indicates significant association (p-value < 0.05); **: Indicates highly significant association (p-value <0.01).

# Discussion

In this study, the prevalence of hypertension among zonal civil servants was 24.5 %. Old age, male sex, current khat chewing, current moderate alcohol drinking, obesity and central obesity were found to be risk factors for hypertension.

In the current study, the overall prevalence of hypertension was 24.5% which is consistent with other studies conducted in Bahir Dar city (25.1%) and in Addis Ababa (25%) [22, 23]. However, this finding is lower than study findings in Gondar city (28.3%) and Jigjiga town (28.3%) [24, 25]. The reason for these discrepancies might be due to all study participants in Gondar city were age 35 and above, and the study participants in Jigjiga town were age 25 to 65 years old, whereas the participants in this study were age 18 years and above. Different studies indicate that the prevalence of hypertension increases in older age [16, 17, 22, 24, 26-30]. The other potential reason for these difference might be the study settings. Both previous studies were community based, whereas our study is institution based.

However, the prevalence of this study is higher than that of community-based studies conducted in Bedele town (16.9%) and Durame town (22.4%) [30, 31]. Similarly, hospital-based study conducted in Jimma showed lower prevalence (13.2%) than the current study [32].

These variations might be due to the first two studies were community-based in which the study participants were with different types of occupation including farmers and daily laborers, whereas in this study the participants were civil servants. The participants of the study in Jimma were patients who came from rural and urban areas but the participants in this study were from urban setting which might explain the discrepancy, at least partly. The population in our study was civil servants with more sedentary life style related to their work condition, which puts the

population addressed by the present study at a higher risk than that of the population in previous studies. The magnitude of hypertension is structurally higher in urban than in rural settings mainly because of contextual and behavioral factors associated with urban environments such as dietary changes and sedentary lifestyle that together form a complex system conducive for developing hypertension [33].

Different studies have showed that several factors have significant association with hypertension. In this study, the odds of hypertension in males was more than four-fold higher as compared to the females. This is similar with several previous studies conducted in different areas. The study conducted in Tigray among civil servants showed the odds of hypertension in males to be two times higher as compared to the females [29], similarly to the studies among students of Gondar University [34] and civil servants in Addis Ababa [28]. Similarly, studies conducted in China, India and Nepal revealed similar findings [35-37]. The reason for this difference might be due to the exposure towards different behavioral risk factors for hypertension, which is higher among males than females in most areas. Moreover, the molecular mechanisms underlying vasculature, nervous system, and kidney functions, which lead to hypertension and the pathways for the control of blood pressure may explain the differences between the sexes [38]. In contrast, some studies from Sub-Saharan Africa the prevalence of hypertension was higher in females than males [39].

The current study also revealed that old age was significantly associated with hypertension. Similar findings were reported from the study conducted at Hawassa University [27], in Tigray [29], Addis Ababa [17], and Bahir Dar city [22], Durame town [30], Gondar city [24], Jmma town [40], and Bedele town of Ethiopia [31]. Similarly, studies conducted in US National Health and Nutrition Examination Survey and China coincided with the current study [26, 35]. This

might be due to the physiological change of blood vessels as the individual's age is increased, in which blood vessels might lose flexibility due to hardening of the arteries as age advances [41].

Current alcohol drinking was significantly associated with hypertension. Studies conducted in Addis Ababa [17], Gondar city [24], Jimma town [40] showed similar significant association. Likewise, studies conducted in Ghana [42], Nepal [37], and India [36] also showed significant association between alcohol drinking and hypertension. This might be due to alcohol consumption raises the amount of lipids in the bloodstream, which can damage the arteries. This in turn leads to hardening of arteries that increases the risk of blood clots which can raise blood pressure. Another reason might be alcohol has high calories and sugar, which can increase the risk of high blood pressure in the long term by adding to the body fat [43].

In this study, BMI $\geq$ 30 $Kg/m^2$ was significantly associated with hypertension. Similar findings reported from studies in Hawassa University [27], Tigray region [29] and Addis Ababa [17]. Studies conducted in Indian and Nepal also reported consistent findings [36, 37]. The reason for this might be additional fat or adipose tissue in the body need oxygen and nutrients in order to live, which requires the blood vessels to circulate more blood to the fat tissue. Moreover, blood flow in the heart, kidneys, gastrointestinal tract, and skeletal muscle also increases with weight gain due to increased metabolic rate and growth of the organs and tissues in response to their increased metabolic demands, this in turn increases pressure on the walls of the artery [44].

The result of this study also indicated that central obesity had an association with hypertension. This finding agreed with studies conducted in Bedele town [31], Addis Ababa [12] and India [36]. This might be resulted from increased pressure in the abdominal cavity due to central obesity. When the intra-abdominal pressure does rise, the pressure in the veins of the legs must rise above the abdominal pressure before the abdominal veins will open and allow the blood to

flow from the legs to the heart. Thus, if the intra-abdominal pressure is +20 mm Hg, the lowest possible pressure in the femoral veins is also about +20 mm Hg [44].

Moreover, this study showed that current khat chewing was significantly associated with hypertension. Studies conducted in Jimma and Gondar also reported a significant association between khat chewing and hypertension [16, 45]. The possible reason for this may be cathinone (a stimulant chemical in khat) increases blood pressure through noradrenaline release which is similar to amphetamine to produce vasoconstriction [46].

In conclusion, one in four civil servants in study area are hypertensive. Male sex, old age, current moderate alcohol consumption, current khat chewing, obesity and central obesity were the risk factors for hypertension among civil servants in Sidama Zone departments. Local health officials and other stakeholders should work on civil servants towards awareness creation regarding hypertension and promotion of healthy life styles including weight management, regular physical activity, and avoiding harmful use of alcohol and khat.

**Consent for publication**

As there were no images and videos associated with this study participants, only informed consent was held.

**Supplementary Materials**

Data will be available upon request from the corresponding author.

**Conflict of Interest**

The study was conducted solely on an academic basis and the authors declare that they have no competing interests.

**Acknowledgements**

The authors thank the School of Public Health of Hawassa University for providing oversight for the conduct of the study. The study participants also deserve thanks for willfully taking part in the study. Finally, the authors are grateful for Sidama Zone Health Department for providing support during data collection.

**Authors' contributions**

Conceptualization: BB, AA. Data curation: BB. Formal analysis: BB, AY, AA. Investigation: BB, AA. Methodology: BB, AA. Project administration: BB. Resources: BB. Software: BB, AY, AA. Supervision: BB, AA. Validation: BB, AY, AA. Visualization: BB, AY, AA. Writing - original draft: BB, AY. Writing-review & editing: AA.

## References


1. World Health Organization (WHO). Global Status Reoprt on noncommunicable diseases. Geneva, Switzerland: 2014.

2. Lim SS, Vos T, Flaxman AD, Danaei G, Shibuya K, Adair-Rohani H, et al. A comparative risk assessment of burden of disease and injury attributable to 67 risk factors and risk factor clusters in 21 regions, 1990-2010: a systematic analysis for the Global Burden of Disease Study 2010. Lancet 2013;380(9859):2224-60.

3. World Health Organization (WHO). A global brief on Hypertension, Silent killer, global public health crisis, World Health Day. Geneva, Switzerland: WHO, 2013.

4. Levi MN, Macquin-Mavier I, Tropeano AI, Bachoud-Levi AC, Maison P. Antihypertensive classes, cognitive decline and incidence of dementia: a network meta-analysis. J Hypertens. 2013;31(6):1073-82.

5. Khan KS, Wojdyla D, Say L, Gülmezoglu AM, Van Look PF. WHO analysis of causes of maternal death: a systematic review. Lancet. 2006;367(9516):1066–74.

6. NCD Risk Factor Collaboration (NCD-RisC). Worldwide trends in blood pressure from 1975 to 2015: a pooled analysis of 1479 population-based measurement studies with 19.1 million participants Lancet 2017;389(10064):37–55.

7. Forouzanfar MH, Liu P, Roth GA, Ng M, Biryukov S, Marczak L, et al. Global burden of hypertension and systolic blood pressure of at least 110 to 115 mmHg 1990 - 2015. JAMA. 2017;317(2):165-82.

8. WHO. Global status report on noncommunicable diseases 2010 Geneva: Switzerland, World Health Organization: 2011.

9. Gaziano TA, Bitton A, Anand S, Weinstein MC. The global cost of nonoptimal blood pressure J Hypertens. 2009;27(7):1472–7.

10. A. Tran, B. Gelaye, B. Girma, S. Lemma, Y. Berhane, T. Bekele, et al. Prevalence of Metabolic Syndrome among Working Adults in Ethiopia. International Journal of Hypertension. 2011:8.

11. Kibret KT, Mesfin YM. Prevalence of hypertension in Ethiopia: a systematic meta-analysis. Public Health Reviews 2015;36(14):12.

12. Tesfaye F, Byass P, Wall S. Population based prevalence of high blood pressure among adults in Addis Ababa: uncovering a silent epidemic. BMC Cardiovasc Disord. 2009;9(39):1-10.

13. Steven van de V, Hilda A, Samuel O, Ademola O, Charles A, Isabella A, et al. Status report on hypertension in Africa - Consultative review for the 6th Session of the African Union Conference of Ministers of Health on NCD's. PanAfrican Medial J. 2013;16:38.

14. Ethiopian Public Health Association. Emerging Public Health Problems in Ethiopia: Chronic Non-Communicable Diseases 2012. 117 p.

15. Guchiye B, Enquselsies F. Prevalence and Associated Factors of Hypertension among workers of Steel factories, Akaki, Addis Ababa. AAU Institutional Repository. 2014;49:52.

16. Abebe SM, Berhane Y, Worku A, Getachew A. Prevalence and Associated Factors of Hypertension: A Crossectional Community Based Study in Northwest Ethiopia. PLoSONE. 2015;10(4):11.

17. Kassawmar A, Abel FD, Kefyalew AA. Prevalence of hypertension among federal ministry civil servants in Addis Ababa, Ethiopia: a call for a workplace-screening program. BMC Cardiovasc Disord. 2015;15(76):1-6.

18. WHO. The World Health Organization STEPwise approach to chronic disease risk factor surveillance (STEPS) Geneva, Switzerland, WHO:2015. p. 14.

19. Bryan W, Giuseppe M, Wilko S, Enrico AR, Michel A, Michel B, et al. 2018 ESC/ESH Guidelines for themanagement of arterial hypertension. European Heart Journal 2018;39(33):1–98.

20. State of Alaska. State of Alaska Measuring Height/Weight and Calculating BMI Guidelines for Schools. Department of Health and Social services. Alaska2012. p. 1-26.

21. WHO. Waist Circumference and Waist-Hip Ratio Report of a WHO Expert Consultation. Geneva: WHO: 2008.

22. Anteneh ZA, Yalew WA, Abitew DB. Prevalence and correlation of hypertension among adult population in Bahir Dar city, northwest Ethiopia: a community based cross-sectional study. International Journal of General Medicine. 2015;8:175-85.

23. Abdissa SG, Feleke Y, Awol M. Prevalence of hypertension and pre-hypertension in Addis Ababa, Ethiopia: A survey done in recognition of World Hypertension Day, 2014. Ethiop J Health Dev 2015;29(1):22-30.

24. Awoke A, Awoke T, Alemu S, Megabiaw B. Prevalence and associated factors of hypertension among adults in Gondar, Northwest Ethiopia: a community based cross-sectional study. BMC Cardiovasc Disord. 2012;12(113).

25. Asresahegn H, Tadesse F, Beyene E. Prevalence and associated factors of hypertension among adults in Ethiopia: a community based cross‑sectional study. BMC Res Notes 2017 10(1):629-37.

26. Yoon SS, Fryar CD, Carroll MD. Hypertension Prevalence and Control Among Adults: United States, National Health and Nutrition Examination Survey 2011–2014. NCHS Data Brief 2015:(220):1-6.

27. Esaiyas A, Teshome T, Kassa D. Prevalence of Hypertension and Associate Risk Factors among Workers at Hawassa University, Ethiopia: An Institution Based Cross Sectional Study. J Vasc Med Surg 2018;6(1):354-60.

28. Fikadu G, Lemma S. Socioeconomic Status and Hypertension among Teachers and Bankers in Addis Ababa, Ethiopia. . International Journal of Hypertension. 2016:7.

29. Bayray A, Meles KG, Sibhatu Y. Magnitude and risk factors for hypertension among public servants in Tigray, Ethiopia: A cross-sectional study. PLoS ONE 2018;13(10):1-10.

30. Helelo TP, Gelaw YA, Adane AA. Prevalence and Associated Factors of Hypertension among Adults in Durame Town, Southern Ethiopia. PLoS ONE 2014;9(11):1-9.

31. Bonsa F, Gudina EK, Hajito KW. Prevalence of hypertension and associated factors in Bedele town, Southwest Ethiopia. (DOI: http://dx.doi.org/10.4314/ejhs.v24i1.3). Ethiop J Health Sci 2014;24(1):21-6.

32. Gudina EK, Michael Y, Assegid S. Prevalence of hypertension and its risk factors in southwest Ethiopia: a hospital-based cross-sectional survey. Integrated Blood Pressure Control. 2013;6:111-7.

33. Agyemang C. Rural and urban differences in blood pressure and hypertension in Ghana, West Africa. public health. 2006;120(6):525-33.

34. Tadesse T, Alemu H. Hypertension and associated factors among university students in Gondar, Ethiopia: a cross-sectional study BMC Public Health 2014;14(937):1-6.

35. Gao Y, Chen G, Tian H, Lin L, Lu J, Weng J, et al. Prevalence of Hypertension in China: A Cross-Sectional Study. PLoS ONE. 2013;8(6):1-9.

36. Shikha S, Ravi S, Gyan PS. Prevalence and Associated Risk Factors of Hypertension: A Cross-Sectional Study in Urban Varanasi. International Journal of Hypertension. 2017:1-10.

37. Mahendra G, Sanjana Z, Ariful BC, Shaikh AS, Ahsan GU, Mohammad Delwer HH. Prevalence and Associated Factors of Hypertension among Nepalese Civil Servants. . Medical Journal of Clinical Trials & Case Studies. 2018;2 (10):1-10.

38. Zimmerman MA, Sullivan JC. Hypertension: what's sex got to do with its Physiology? . Physiology (Bethesda). 2013;28(4):234-44.

39. Opie LH, Seedat YK. Hypertension in sub-Saharan African populations 2005. Circulation. 2005 112(23):3562-8.

40. Sisay Bissa J, Muktar Beshir A. Prevalence of Tobacco Consumption, Alcohol, Khat (Catha Edulis) Use and High Blood Pressure among Adults In Jimma Town, South West Ethiopia Science Journal of Public Health. 2015;3(5):650-4

41. Kotcher T. Hypertensive vascular disease. In: Athonys F, Eugene B, Dennis K (eds). Harrison's Principles of internal medicine USA: McGraw-Hill Companies, 2011, 18th ed.2:1549–62.

42. Bosu WK. Epidemic of hypertension in Ghana: a systematic review. BMC Public Health 2010;10(418):1-14.

43. WHO. Global status report on alcohol and health. Geneva: World Health Organization 2018.

44. Jones CE, Coleman TG. Guyton and Hall Textbook of Medical Physiology. Circulatory physiology: Vascular Distensibility and functions of the Arterial and Venous systems. cardiac output and its regulation. Philadelphia: WB Saunders, 2011, 12th ed. page: 172-226.

45. Sisay Bissa AM, Teshome Gobena. prevalence of hypertension and its association with substance use among adults living in jimma town, south west ethiopia. 2012:11.

46. Hassan NA, Gunaid AA, Abdo-Rabbo AA, Abdel-Kader ZY, al-Mansoob MA, Awad AY, et al. The effect of Qat chewing on blood pressure and heart rate in healthy volunteers. Trop Doct. 2000;30(2):107-8.
